# Bacterial Membrane Vesicles for In Vitro Catalysis

**DOI:** 10.3390/bioengineering10091099

**Published:** 2023-09-20

**Authors:** Meghna Thakur, Scott N. Dean, Julie C. Caruana, Scott A. Walper, Gregory A. Ellis

**Affiliations:** 1 College of Science, George Mason University, Fairfax, VA 22030, USA; 2 Center for Bio/Molecular Science and Engineering, Code 6900, U.S. Naval Research Laboratory, Washington, DC 20375, USA; 3 American Society for Engineering Education, Washington, DC 20036, USA

**Keywords:** outer membrane vesicles (OMVs), biocatalysis, biomanufacturing

## Abstract

The use of biological systems in manufacturing and medical applications has seen a dramatic rise in recent years as scientists and engineers have gained a greater understanding of both the strengths and limitations of biological systems. Biomanufacturing, or the use of biology for the production of biomolecules, chemical precursors, and others, is one particular area on the rise as enzymatic systems have been shown to be highly advantageous in limiting the need for harsh chemical processes and the formation of toxic products. Unfortunately, biological production of some products can be limited due to their toxic nature or reduced reaction efficiency due to competing metabolic pathways. In nature, microbes often secrete enzymes directly into the environment or encapsulate them within membrane vesicles to allow catalysis to occur outside the cell for the purpose of environmental conditioning, nutrient acquisition, or community interactions. Of particular interest to biotechnology applications, researchers have shown that membrane vesicle encapsulation often confers improved stability, solvent tolerance, and other benefits that are highly conducive to industrial manufacturing practices. While still an emerging field, this review will provide an introduction to biocatalysis and bacterial membrane vesicles, highlight the use of vesicles in catalytic processes in nature, describe successes of engineering vesicle/enzyme systems for biocatalysis, and end with a perspective on future directions, using selected examples to illustrate these systems’ potential as an enabling tool for biotechnology and biomanufacturing.

## 1. Introduction

In the most basic terms, biocatalysis is a chemical process in which biomolecules such as enzymes perform reactions between organic components. Biocatalysis encompasses numerous technologies, from small scale to industrial scale, and from in vitro reactions with only enzyme catalysts, substrates, co-factors, and buffer, to fermentations using whole-cell microbial cultures. Backed by advances in synthetic biology that have enabled substantial microbial engineering, biocatalysis is emerging as a competitor to classical chemical routes of synthesis for specialty chemicals, therapeutics, and novel materials.

Enzyme-based catalysts can offer numerous advantages over conventional chemical catalysts, including higher efficiency, tremendous specificity (allowing even synthesis of chiral products that are challenging or impossible using classical synthesis methods), and the potential for customization of protein function using molecular biology techniques [1]. There are also benefits in terms of environmental concerns, as bioproduction is performed under mild physiological conditions (temperature, pressure, pH, etc.) as compared to traditional chemical synthesis and often with by-products that are inert or pose less of a challenge for neutralization and disposal. This biotolerance also pertains to the catalysts themselves, which are similarly biodegradable, unlike many conventional chemical catalysts.

One of the major strategies for biocatalysis is the use of enzymes in whole-cell microbial cultures. There are advantages in terms of cost, as growth of commonly used chassis organisms such as *Escherichia coli* is generally not cost-prohibitive, and the use of whole cells removes the cost in money and time for individual purification of each enzyme required for a desired process. Cells can often be used repeatedly, while the longevity of purified enzymes is more limited and they sometimes cannot be separated in usable form from reaction products once synthetic steps are complete [2]. Containment within a living cell provides conditions to protect enzymes, supply cofactors that would otherwise be costly to produce and supply externally, and maintain compartmentalization and proximity of enzymes to maximize the flow of intermediates from one reaction step to the next.

Advances in our understanding of cellular metabolism, protein function, and gene regulation and the development of corresponding synthetic biology tools to manipulate these systems allow for detailed fine-tuning of a desired metabolic pathway for biocatalysis [3,4]. Non-native enzymes from diverse sources including bacteria, plants, and animals can be expressed in microbial chassis organisms including *E. coli*, *Bacillus subtilis*, *Saccharomyces cerevisiae*, and others using established and well-described protocols and systems for scalable production [4]. Biosynthetic pathways can be assembled from a single source organism though the construction of a de novo pathway comprised of enzymes from multiple species optimized for specific biophysical properties; this can lead to significant improvements in reaction stability, speed, and efficiency [2,5]. One of the more successful examples of this is the bioproduction of the antimalarial artemisinin. Originally identified in the plant *Artemisia annua*, researchers invested heavily in developing methods of mass producing this high-demand anti-malarial heterologously to meet demand. In a multitude of studies highlighted in a review by Muangphrom et al., recombinant expression of the *A. annua* biosynthetic pathway was augmented with enzymes from a variety of plant, fungal, and microbial species to create a highly efficient production system in microbes such as *E. coli* and *S. cerevisiae* [6].

Biomanufacturing in microbial chassis is not without its limitations, however (see review by Chen for additional descriptions [7]). Much like a chemical reaction that is poisoned by the formation of certain byproducts, substrate diffusion, and other reaction variables, biomanufacturing processes can be “killed” by changes in the reaction environment, whether this is the intracellular environment or the culture conditions themselves [8]. Researchers have identified and continue to explore methods of improving biomanufacturing capabilities including strain engineering, optimizing fermentation, and continuous monitoring, among others [7,9,10]. In addition to process and strain engineering, other enabling paths to enhance biomanufacturing capabilities are also being explored. In vitro biosynthetic pathways using a number of platforms including purified enzymes, scaffolded (or immobilized) enzymes, and cell-free lysate-based systems have been explored as alternate or enabling systems [11,12,13]. These systems are able to bypass issues with toxicity of chemical components to a cellular host but can suffer from poor stability and longevity due to the absence of biological components that come with cell-based systems. An emerging capability in biomanufacturing, and the subject of this review, involves the use of a naturally occurring cellular process that can be engineered to enable the one-pot production of biological catalysts and that encompasses advantages of both cell-based and in vitro biocatalysis.

Most bacteria studied to date release nanosized proteoliposomes from their outermost membrane, referred to as membrane vesicles (MVs) or outer membrane vesicles (OMVs) [14]. In nature, these biological nanoparticles serve a number of potential roles that are actively studied, including pathogenesis, cellular defense, cellular signaling, and others [15]. Although the language for describing bacterial MVs varies between articles in the literature, MVs most commonly refer to vesicles that bleb from the membrane of Gram-positive bacteria or fungi, while OMVs suggest vesicles derived from the outer membrane of Gram-negative bacteria, and the term extracellular vesicles is reserved for vesicles that bleb from the surface of eukaryotic cells [16]. In bacteria, the blebbing of vesicles from Gram-negatives and Gram-positives primarily occurs through two distinct pathways: (1) the blebbing of membrane material from living bacteria and (2) an endolysin-triggered process that allows for vesicle passage through a degraded peptidoglycan layer. MVs/OMVs and the mechanisms of blebbing are extensively covered in a recent review [16]. As will be discussed in the subsequent sections, OMVs and MVs can also exhibit catalytic activity under some circumstances as microbial enzymes are localized to OMVs and then OMVs are released into the environment. This phenomenon, in conjunction with advances in synthetic biology, has allowed researchers to design and assemble their own catalytic systems through careful engineering of cellular systems to control localization of target enzymes to OMV/MVs as they form [17,18,19].

**Figure 1:**
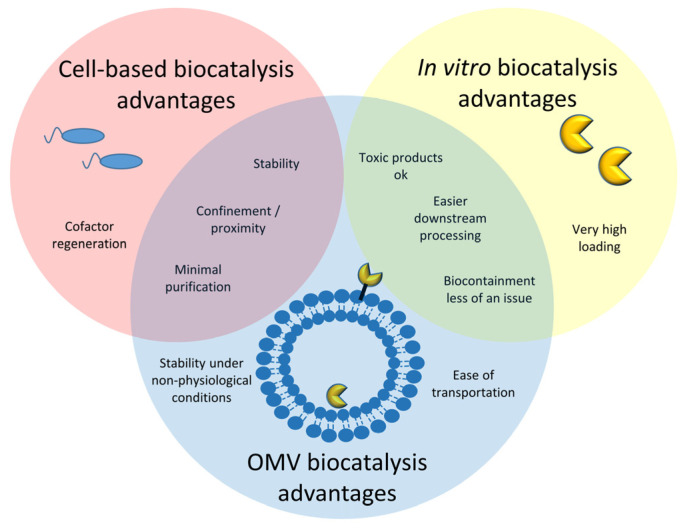
Bacterial membrane vesicles provide advantages of both cell-based biocatalysis and biocatalysis using purified enzymes.

As alluded to above, OMV biocatalytic systems allow advantages of both cell-based and purified enzyme in vitro biocatalysis (Figure 1). Similar to cells, OMVs have the option to allow enzymes to remain encapsuled and therefore in a cellular context, which may stabilize the enzymes, or displayed on the surface, which may help facilitate access to substrates or release of product. Multiple enzymes can be packaged into or onto an OMV, allowing for the maintenance of confinement and/or proximity. OMVs with multiple enzymes also only require one purification of the entire OMV package instead of multiple purifications of multiple individual enzymes. Similar to in vitro systems, components of a reaction pathway that would be toxic to a host organism are permissible. Purification of the OMVs away from other cellular components and/or media can facilitate downstream processing of reaction products. Finally, since OMVs are non-living, biocontainment becomes less of an issue. In this review, we will describe some of the natural catalytic systems observed within OMVs/MVs, describe several elegant systems for assembling catalytic systems within OMVs to build cell-free catalytic systems, and finally give a perspective on future directions for addressing some of the challenges and opportunities in the field. These examples are not meant to be exhaustive, but to give illustrative examples of some of these advantages of OMV biocatalytic systems to inspire their future development and use.

## 2. Bacterial Membrane Vesicles in Biocatalysis—OMVs Naturally Involved in Catalysis

Without intervention by engineering, MVs/OMVs produced by certain bacteria have been demonstrated to catalyze a variety of vital reactions through the enzymes they naturally carry. Much of the extant literature on the use of natural MVs/OMVs for enzyme catalysis revolves around the breakdown of lignocellulosic biomass, or dry plant matter, as well as other complex polysaccharides (Table 1). Since lignocellulose is the most abundant material on Earth that can be used for biofuels production, such as bioethanol, catalysis involved in its processing has garnered particular interest in a variety of fields [20]. The ability to degrade lignocellulose fibers into their composite parts in a financially feasible manner has several barriers that must be overcome. One barrier results from the chemical pretreatment processes generally used to prepare the lignocellulosic biomass for downstream processing, the toxic side products of which, such as furfural, can be strong inhibitors of cellular growth at low concentrations [21], necessitating a cell-free system if a biotechnology-based solution is to be used downstream of these pretreatments.

**Figure 2:**
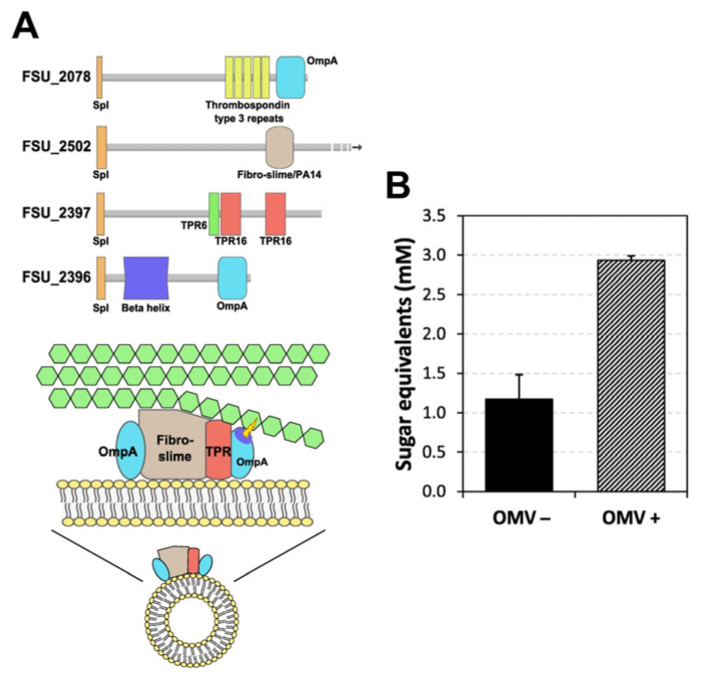
OMVs from the cow rumen symbiote *Fibrobacter succinogenes* contain various proteins and enzymes to help degrade plant fibers. (**A**) Possible mode of action of a complex of proteins and enzymes on OMVs: two OmpA family proteins (could hydrolyze substrate), a fibro-slime protein (helps bind cellulose) [32], and a TPR (tetratricopeptide repeat) domain protein (acts as protein scaffold) [33]. (**B**) Switchgrass was pretreated with or without OMVs and then subjected to enzymatic degradation by Celluclast and Novozym 188; 2.4× more sugar was released using OMV pretreatment conditions. Adapted with permission from Ref. [22]. Copyright 1999–2023 John Wiley & Sons, Inc. All rights reserved.

Recently, OMVs that degrade lignocellulosic biomass and that are produced by important residents of the rumen microbiota have been discovered [31]. It is thought that over millennia, symbiotic gut and rumen microbiota and their hosts have co-evolved these systems to convert consumed plant fibers into a carbon and energy source. Arntzen et al. showed that *Fibrobacter succinogenes*, a cellulose-degrading species found in the cow rumen, produces OMVs that have several different classes of proteins that enable efficient degradation of plant fibers, including fibro-slime proteins, cellulases and hemicellulases that can degrade cellulose, pectin, and hemicelluloses. Importantly, the group reported a significant 2.4-fold increase in sugar equivalents resulting from switchgrass that was pretreated with *F. succinogenes* OMVs [22], relative to the cellulase-only control (Figure 2). Biologically, the group hypothesized that the OMVs may enable *F. succinogenes* to have improved accessibility to the nutrient-rich components of lignocellulose following OMV-led degradation. Interestingly, although *F. succinogenes* itself only utilizes cellulose as a carbon source, the OMVs it produces contain a cocktail of polysaccharide-degrading enzymes, suggesting a supportive role of its OMVs for other organisms, including the host, in the rumen microbiota [22]. In another more recent study, Ichikawa et al. found the Gram-positive cellulolytic *Clostridium thermocellum* also produces membrane vesicles that degrade cellulose. However, in the case of *C. thermocellum*, the group determined via immunoelectron microscopy that the carbohydrate-active enzyme complexes (or cellulosomes) are localized to the outer vesicle surface [23].

**Figure 3:**
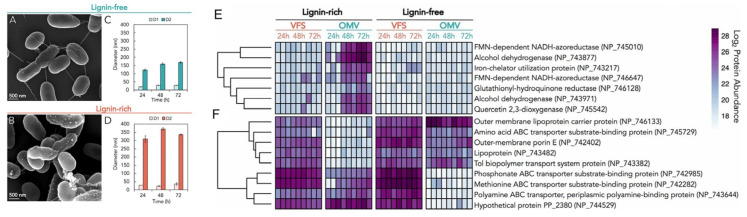
OMVs from the saprotrophic soil bacterium *Pseudomonas putida* KT2440 can break down lignin. (**A**–**D**) The bacterium produces two different sizes of OMVs when in lignin-rich media (**E**,**F**). Specific components of the exoproteome are selectively incorporated into OMVs versus the vesicle-free secretome (VFS). (**E**) shows proteins with known or putative functions in oxidation–reduction processes. (**F**) shows proteins with known or putative functions as lipoprotein, small molecule binding protein, transport-related, or outer membrane porins. Adapted with permission from Ref. [24]. Copyright 2020 reference authors.

Beyond polysaccharides, other polymers within plant biomass, including lignin, a class of cross-linked phenolic polymers, are difficult to break down for nutrient sources or other uses. While lignin had previously been shown to be depolymerized in nature by various fungal species, recently Salvachúa et al. showed that bacterial OMVs, too, can break down lignin [24]. By conducting proteomic analysis under various conditions of the saprotrophic soil bacterium *Pseudomonas putida* KT2440, they showed that when grown in lignin-rich media, putatively ligninolytic enzymes such as de-colorizing peroxidases are significantly enriched in the exoproteome relative to intracellular proteins. In addition, they identified other enzymes that they hypothesize modify both oligomeric and monomeric aromatic or phenolic compounds, including a 2,3-quercetin dioxygenase, a xenobiotic reductase, and azoreductases, each of which was enriched in the *P. putida* exoproteome in lignin-rich media. Regarding OMVs, they showed that two distinct populations of OMVs develop in lignin-rich media—as determined by diameter measurements with transmission electron microscopy, suggesting a large corresponding phenotypic shift by *P. putida* (Figure 3A–D). They also show that several proteins known or putatively thought to be involved in oxidation–reduction processes are enriched in OMVs over the vesicle-free secretome (VFS) when grown in lignin-rich media (Figure 3E), including the FDH-dependent NADH-azoreductase and the quercertin 2,3-dioxygenase, as well as other proteins which are differentially expressed in OMVs in lignin-free versus lignin-rich media (Figure 3F). The authors note that *P. putida* was previously known to be well-suited for bioremediation with the documented ability to degrade toluene and other toxic pollutants, potentially using the same versatile phenolic compound-degrading enzymes with activity in lignin catabolism, suggesting *P. putida* OMVs have potential as tools in synthetic biology and biotechnological applications beyond improving microbial lignin conversion in biotechnology venues [24]. Another species, *P. capeferrum,* has recently been shown to biodegrade extracellular polyurethane via OMVs where the degradation potentially involves both periplasmic as well as membrane-bound hydrolases [25].

Similar to the rumen microbiota discussed above, the human gut microbiota has also symbiotically co-evolved systems to convert consumed foodstuffs into a carbon and energy source. Previously, Rakoff-Nahoum et al. demonstrated that OMVs produced by a number of *Bacteroides* species can break down complex polysaccharides, including the fructose polymers inulin and levan, the xylose polymer xylan, and the glucose polymer amylopectin. The breakdown of these carbohydrates promoted growth of other bacterial species unable to degrade those polysaccharides and potentially provided beneficial material to the host [26]. This study, however, did not identify the particular enzymes responsible for these actions. More recently, Elhenawy et al. reported that two carbohydrate-digesting *Bacteroides* species (*B. fragilis* and *B. thetaiotaomicron*) that inhabit the human gut produce fibrolytic OMVs [27]. In this study, through proteomic analysis, the authors showed that *Bacteroides* preferentially packages a large number of hydrolases in OMVs, many of which were detected exclusively in vesicles as opposed to the bacterial outer membrane. Specifically, they identified xylanase, β-xylosidase, glycosyl hydrolase, chitinase, among others, exclusive to *Bacteroides* OMVs, targeting glycans that are not substrates for human hydrolases, but the products of some of which can be utilized by both microbiota members and are beneficial for the host [27]. These *Bacteroides* OMVs may see application outside of the human gut with particular interest in degradation of polymers to D-xylose, where D-xylose works as a feedstock for biosynthetic cascades for other organisms [34].

Shifting to the marine environment, several recent studies have begun to investigate the role of OMVs produced by marine bacteria such as *Vibrio harveyi* [35,36]. Reported to be very prevalent, Lynch and Alegado estimate that ~1,000,000 metric tons of protein are released in the ocean via OMV blebbing every day [36]. Investigating this large previously unaccounted-for biomass in the marine environment, researchers have noted that the ecological impact of bacterial OMVs include the degradation of seaweed plant material. In a recent proteomics study, Naval et al. showed that a significant proportion of the proteins contained within the membrane vesicles secreted by the seaweed-associated bacterium, *Alteromonas macleodii* KS62, were hydrolytic enzymes—approximately 30% [28]. Of particular interest were glycoside hydrolases, which they found were the responsible constituent of the OMVs for hydrolyzing κ-carrageenan, the most prominent polysaccharide composing the cell wall of seaweeds like *Kappaphycus*, a genus of red algae. After 12 h of incubation of *Kappaphycus* seaweed biomass with *A. macleodii* OMVs, the group recorded a >10-fold increase in reducing sugars compared to the OMV-free control. Hypothesized to assist *Alteromonas* to invade and colonize the seaweed biomass, with a potentially large role in this marine environment niche, these OMVs may be of interest for degrading seaweed in biotechnology applications such as bioethanol production and for extracting valuable biomolecules, e.g., carotenoids and vitamins [28]. While not the subject of this review, it is worth noting that in addition to bacteria, fungi have also been shown to produce extracellular vesicles (EVs) carrying lignocellulolytic enzymes. The industrially relevant fungi *Trichoderma reesei* produces more EVs in the presence of cellulose in comparison to glucose and glycerol, and the vesicles had higher β-glucosidase activity than supernatant [29].

While the breakdown of lignocellulose, polysaccharides, and other targets of interest can clearly benefit from a biotechnology solution, in practice many processes require a cell-free system if used with cellularly toxic pretreatments. Here, several recent reports have demonstrated the promise of naturally occurring, non-engineered OMVs in the degradation of plant matter, foodstuffs, and other matter, suggesting that, for certain applications, cell-free catalysis systems are readily discoverable in nature. In some cases, these systems may be directly portable into industrial applications; in others, these systems can instead be targets to be engineered into industrially-used model organisms.

## 3. Engineered Outer Membrane Vesicles for Biocatalysis

Extensive efforts have been made in the field of OMV engineering to enhance their usage in vaccination, tumor therapy, and antibiotic and small molecule delivery [37,38,39]. The focus of this review is the complementary efforts to engineer OMVs for biocatalysis using genetic engineering techniques. Some of the initial efforts in the field of OMV engineering were focused on encapsulation of single proteins/enzymes such as green fluorescent protein (GFP), β-lactamase (Bla) and organophosphate hydrolase (OPH) via fusion to the native vesicle-associated, pore-forming cytotoxin ClyA of *E. coli* [40,41]. Several other approaches have been described for localization of enzymes on the surface or in the lumen of OMVs, such as fusion with the autotransporter hemoglobin protease Hbp and the outer membrane protein OmpA, to name a few [42,43,44]. Efforts later expanded to the association of multiple enzymes of a pathway with OMVs (see below). Regarding the type of catalyzed reactions, OMVs have been engineered to encapsulate single enzymes or enzymatic pathways for the degradation of substrates such as cellulose, organophosphates, and coelenterazine analogs, among other uses (Table 2). Below, we highlight some of these examples to emphasize the possibilities of engineering OMVs for biocatalysis.

### 3.1. Cellulose Hydrolysis

**Figure 4:**
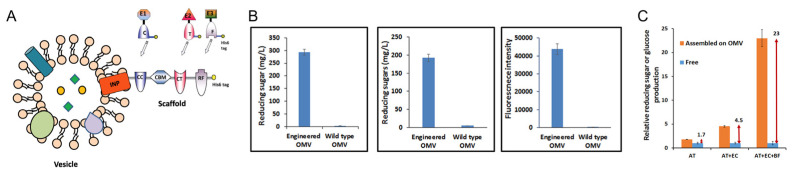
Engineered OMVs with scaffolded enzymes for cellulose degradation. (**A**) OMVs were engineered with a scaffold fused to ice nuclear protein (INP) containing three cohesion domains (CC, CT, and RF) and a cellulose-binding module (CBM). Three cellulases (E1, E2, E3) were fused with the corresponding specific dockerin domains (C, T, F) to enable ordered assembly. B. The bacterium produces two different sizes of OMVs when in lignin-rich media. (**B**) Binding of each cellulase-dockerin fusion to engineered OMVs as shown by activity assays, left to right = AT (endoglucanase fused to DocT), EC (exoglucanase fused to DocC), and BC (β-glucoside fused to DocF). (**C**) All three cellulase-dockerin fusions scaffolded and assembled on OMVs led to a 23× increase in sugars relative to the same amount of free enzymes. Adapted with permission from Ref. [19]. Copyright 2014 reference authors.

As described above, one of the natural targets of OMV catalysis is the degradation of complex polymers such as cellulose. A key example of how this has been engineered into the model organism *E. coli* was described by Park et al. [19]. The authors recruited multiple enzymes constituting a multienzyme complex known as the cellulosome to OMVs. Cellulosomes are naturally produced by anaerobic bacteria for enhanced cellulose hydrolysis. To sequentially assemble these enzymes, the authors employed the use of a cohesion (Coh)—dockerin (Doc) interaction-based protein scaffold (Figure 4). The trivalent protein scaffold contained three orthogonal cohesin domains: DocC (from *C. cellulolyticum*), DocT (from *C. thermocellum*) and DocF (from *R. flavefaciens*), and one cellulose-binding module (CBM); the enzymes in turn were fused to corresponding docterin domains. The scaffold was tethered onto the surface of OMVs utilizing a truncated ice nucleation protein anchoring motif (INP). This engineered system led to a 23-fold enhancement in glucose production over the expression of non-complexed enzyme, demonstrating the power of scaffolded multienzyme pathways on OMVs.

### 3.2. Bioremediation

Due to their ease of manufacture and range of toxicities, organophosphate (OP) compounds are widely employed as agricultural pesticides and maintained as chemical warfare agents by several nations [51]. Yet, due to this toxicity, OPs contribute to numerous cases of poisoning and death each year. Conventional methods of decontamination include both physical (removal, dilution, incineration, etc.) and chemical methods which show varying degrees of effectiveness and toxic by-products. Bioremediation of organophosphate compounds have been explored but to date only a single commercial product is available for the detoxification of pesticides [51]. Fortunately, nature has evolved several enzymes capable of degrading OP compounds, such as diisopropylfluorophosphatase (DFPase) from squid, *Loligo vulgaris*, paraoxonase (PON1) from liver, organophosphate hydrolase from *Agrobacterium radiobacter*, and phosphotriesterase (PTE) from *Brevundimonas diminuta* and *Deinococcus radiodurans*. OP toxicity has driven the efforts of numerous research groups to develop sensors, therapeutics, prophylactics, and environmental decontamination tools based on these enzymes [52,53]. Initially, researchers focused on the use of engineered microbes in which an enzyme was localized to the surface of the bacterial cell to generate bioremediation systems [54]. However, the utility of whole cell biocatalysts is limited owing to restrictions on the use of genetically modified organisms by several nations, leading researchers to focus on environmentally friendly cell-free strategies for bioremediation.

One of the initial efforts to engineer OMVs towards this goal was made by DeLisa and coworkers in 2008 where they fused organophosphorus hydrolase to the vesicle-associated toxin ClyA of *E. coli* [40]. This generated synthetic OMVs that were capable of hydrolyzing the organophosphate pesticide paraoxon, with the N-terminal fusion of OPH to ClyA conferring an ~7 fold increase in OPH activity in the OMV compared to the cell surface [40]. This foundational work confirmed the idea that cell-free OMVs could be engineered to degrade OPs in a cell-free manner.

**Figure 5:**
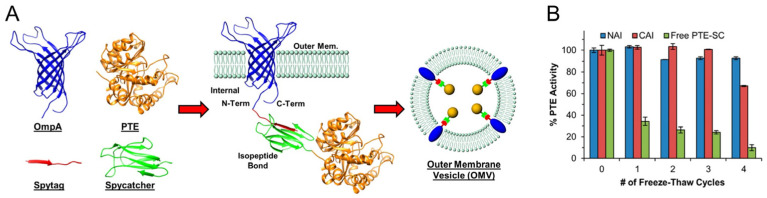
Engineered OMVs for bioremediation using SpyCatcher (SC)-SpyTag (ST) conjugation system. (**A**) Phosphotriesterase fused with SC was conjugated to OmpA fused with ST. (**B**) PTE in engineered OMVs survived multiple freeze–thaw cycles (−80 °C/room temperature) better than free PTE-SC (NAI = ST at N-terminus of OmpA, CAI = ST at C-terminus of OmpA, all with arabinose and IPTG in culture), as indicated by activity using paraoxon as substrate. PDB = 2GE4 (OmpA), 1PTA (PTE), 4MLI (SpyTag), and 4MLI (SpyCatcher). Adapted with permission from Ref. [18]. Copyright 2015 American Chemical Society [58,59,60,61,62,63,64].

Extensive efforts have also been made by the Walper laboratory in the field of OMV engineering for bioremediation. In their initial attempt, the group utilized a SpyCatcher (SC) and SpyTag (ST) based protein–protein interaction system together with the abundant outer membrane protein OmpA of *E. coli* to actively package the phosphotriesterase enzyme PTE from *Brevundimonas diminuta* into OMVs (Figure 5). PTE is a zinc-dependent organophosphate hydrolase that shows high catalytic activity towards a number of pesticides and moderate activity towards nerve agents such as sarin, cyclosarin and VX. The outer membrane protein OmpA fused to ST was utilized to derive the localization of PTE-SC to the vesicles by means of an isopeptide bond formation between OmpA-ST and PTE-SC. This OMV encapsulated PTE-hydrolyzed paraoxon with kinetics comparable to free PTE (indicating that paraoxon could diffuse into the OMVs) and imparted protection to the enzyme to multiple freeze–thaw cycles that otherwise make the free enzyme labile [17]. In a subsequent study, these nanobioreactors were shown to retain substantial activity when subjected to elevated temperature, iterative freeze–thaw cycles and lyophilization [18]. The group was also able to purify PTE-loaded vesicles using immobilized metal-affinity chromatography [55]. For additional information on the methodology of loading OMVs with enzymes such as PTE and purifying enzyme-loaded vesicles, the interested reader is directed to reference [56]. In a more recent study, the PTE OMVs were put to rigorous testing on different environmental water samples and solid surfaces such as glass, painted metal, and fabric. In addition, the PTE-encapsulated OMVs provided protection to the enzyme activity at different pH and high salt conditions. Altogether, these OMV engineering efforts have generated a cell-free bioremediation reagent that can be easily purified, transported in powder form over long distances at room temperature, withstand non-physiological temperature and pH conditions, unlike free enzymes, and can potentially act on different surfaces under extreme environmental conditions [57].

**Figure 6:**
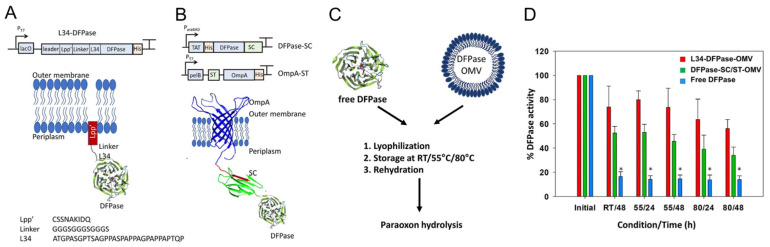
Engineered OMVs using two systems to encapsulate DPFase for bioremediation. (**A**) One strategy for encapsulating DFPase into OMVs was to use lipopeptide Lpp’ as an anchor and fuse this to DFPase with a linker. (**B**) Another strategy was to use OmpA as an answer and conjugate this to DFPase using the SC-ST system. (**C**) Schematic for testing thermal stability of DFPase encapsulated in OMVs using the two strategies compared to free DFPase. (**D**) OMV-encapsulated DFPase was more thermally stable than free DFPase. X-axis numbers are temperature (RT = room temperature) followed by hours of incubation. Error bars are standard deviation (*n* = 3) and * indicates *p* < 0.01 vs. free enzyme. Adapted with permission from Ref. [47]. Copyright 2022 American Chemical Society [59,60,61,62,63,64].

In other work, Su et al. localized OPH and a cellulose-binding domain (CBD) to the surface of an OMV using the ice nucleation protein (INP) [46]. The research team showed that the addition of CBD could allow for a rapid system of OMV purification using an affinity interaction with cellulose. This research group showed similar enhancements in enzyme stability despite the surface localization. More recently, the nerve agent hydrolyzing enzyme DFPase has been packaged into OMVs using two different approaches—the SC/ST approach and a lipopeptide Lpp’ linker-based approach—with the latter leading to enhanced packaging of enzymes into the OMVs (Figure 6) [47,51]. The OMV-encapsulated DFPase hydrolyzed both paraoxon as well as its preferred substrate DFP and also retained activity upon lyophilization. These freeze-dried OMVs had substantial activity upon extended storage at room temperature, and even storage at high temperature led to a minimal reduction in activity, thereby making them an ideal candidate for an environmentally friendly, point-of-need bioremediation reagent.

In addition to organophosphate bioremediation, more recently Woo et al. (2022) have engineered *E. coli* OMVs for bioremediation of antibiotic pollution for the food and agriculture industry by engineering OMVs to encapsulate a class C β-lactamase (CMY-10) from *Enterobacter aerogenes* [45]. The engineered OMVs impressively degraded antibiotics such as nitrocefin and meropenem at a 100–600 fold higher rate than *E. coli*-based whole-cell biocatalysts and exhibited greater catalytic stability compared to free enzymes in the aqueous phase [45]. This is another example of engineered OMVs being inspired by nature, as natural OMVs have previously been shown to carry β-lactamases and hydrolyze β-lactam antibiotics [65].

### 3.3. Biosensing and Bioimaging

**Figure 7:**
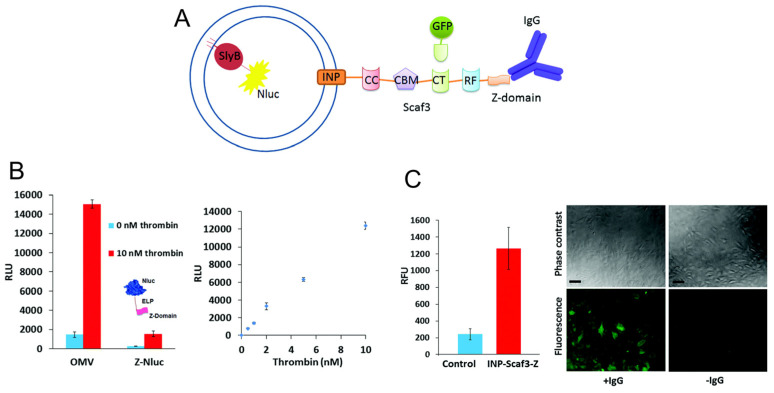
Engineered OMVs using scaffold for biosensing and bioimaging multiple targets. (**A**) Schematic of system. Nanoluciferase (Nluc) is fused to SlyB to anchor inside OMVS. A scaffold with three cohesion domains (CC, CT, and RF) and a cellulose-binding module (CBM) is fused to ice nucleation protein (INP) to display on OMV surface. At the end of this scaffold is an antibody-binding Z-domain that can conjugate to, for example, the IgG antibody displayed. Green fluorescent protein (GFP) is shown fused to a dockerin domain that can bind to a cohesion domain to illustrate the flexibility of the system for detection. (**B**) Thrombin is detected using OMVs as well as a control Z-domain-ELP-NLuc (only 1:1 signal amplification, where ELP = elastin-like-polypeptide moiety). (**C**) Demonstrating the flexibility of this system, HeLa cells were detected by OMVs with the scaffold, Z-domain, and bound GFP with 8× higher fluorescence than control OMVs expressing only SlyB-NLuc. Adapted with permission from Ref. [48] Copyright 2017 Royal Society of Chemistry (https://doi.org/10.1039/C7CC04246A, accessed on 16 June 2023).

Past decades have witnessed a dramatic growth in bioluminescence-based non-invasive molecular imaging due to its ability to circumvent autofluorescence, phototoxicity and photobleaching issues related to fluorescence in addition to low background noise and high sensitivity. While originally renilla and firefly luciferase were widely utilized for luminescence, nanoluciferase (NLuc) has recently gained importance, as it is an ATP-independent luciferase and displays a relatively long half-life, enhanced thermal and pH stability, and lower background. OMVs have been used as a modular platform for biosensing applications via fusion of NLuc on an *E. coli* outer membrane lipoprotein SlyB (Figure 7). In order to facilitate sensing/imaging multiple targets, an antibody-binding Z-domain was simultaneously displayed on the OMV surface via fusion with INP, so that antibodies against various targets could be bound. The Z-domain and INP were separated by a tri-functional cohesion adhesion-based scaffold to enhance functionality (such as the ability to use GFP-dockerin for sensing/imaging as well) [48]. When tested on the analyte thrombin, the detection limit of the OMV sensor was comparable to other reported thrombin detection methods and imparted additional benefits such as ease of preparation and flexibility of electrochemical or colorimetric detection. The same scaffold was also utilized to detect cancer cells by assembly of GFP onto the INP-Scaf3-Z-scaffold and monitoring fluorescence upon addition of anti-MUC1 antibody to HeLa cells.

**Figure 8:**
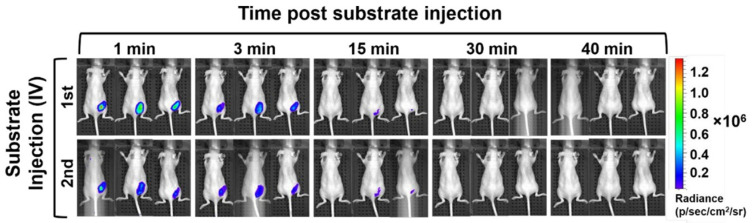
In vivo analysis of engineered OMVs using scaffold for biosensing and bioimaging multiple targets. System is similar to that shown in Figure 7A. Bioluminescence was measured of animals with OMVs subcutaneously injected into right flanks. Substrate as injected intravenously into the tail vail at 0 h (1st) and 0.67 h (2nd) post injection of OMVs. OMVs were able to produce bioluminescence in vivo with OMVs and substrate injected in different locations. Adapted with permission from Ref. [66]. Copyright 2019 American Chemical Society.

The use of NLuc in vivo as a subcutaneous implement can be an extraordinary tool for a variety of biomedical applications and is being investigated with some promising results [49]. Bioengineered OMVs co-expressing ATP-independent NanoLuc luciferase and IgG domain have been used to develop an immunoassay for detection of immunoglobulin IgG which had a comparable detection limit to that of the commercial IgG ELISA kit [49]. In a subsequent study, the multifunctional NLuc-encapsulated OMVs were tested for bioluminescence signals in a murine animal model, thereby exhibiting a strong potential for imaging-related biomedical application (Figure 8) [66]. In vitro cytotoxicity and ex vivo tissue histopathology demonstrated a good biocompatibility of the OMVs. In a more recent study, bioengineered vesicles were employed for optoacoustic imaging via encapsulation of the biopolymer melanin, mediated through expression of tyrosinase, a rate-limiting enzyme in melanin biosynthesis [67]. Although the research on the use of OMVs for biosensing and bioimaging is in its infancy, promising results indicate that they can emerge as valuable tools for these applications in vitro as well as in vivo.

## 4. Other Applications

**Figure 9:**
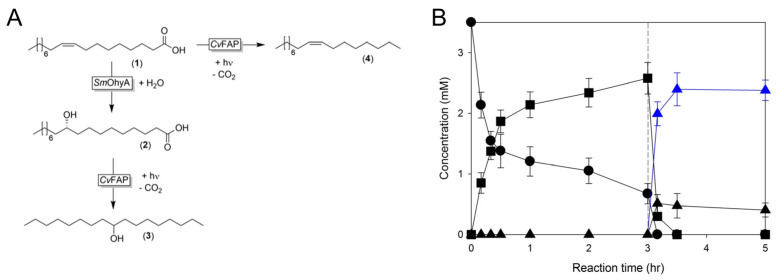
Transformation of unsaturated long-chain fatty acids with OMVs and biocatalysis. (**A**) Reaction scheme using enzymes *Stenotrophomonas maltophilia* fatty acid double-bond hydratase (*Sm*OhyA) and *Chlorella variabilis* NC64A photoactivated decarboxylase (*Cv*FAP). Substrate is oleic acid ((*Z*)-octadec-9-enoic acid, 1), intermediate is (*R*)-10-hydroxyoctadecanoic acid (2), and desired product of the 2-step pathway is 9-hydroxyheptadecane (3). (*Z*)-heptadec-8-ene (4) can be a product of CvFAP or a by-product of the 2-step pathway. (**B**) Reaction of oleic acid using engineered OMVs with *Sm*OhyA and *Cv*FAP. Concentrations of (1, closed circle), (2, closed square), (3, closed blue triangle), and (4, closed triangle) shown. Decarboxylation initiated with blue light at t = 3 h (dashed lines). Experiments were performed in triplicate and error bars are standard deviations. Adapted with permission from Ref. [50]. Copyright 2012 reference authors.

In addition to the above detailed studies, other enzymes have been engineered into bacterial OMVs. Song et al. have engineered *E. coli* OMVs to form nano-scale bioreactors for transformation of unsaturated long-chain fatty acids [50] by packaging the fatty acid double-bond hydratase of *Stenotrophomonas maltophilia* (*Sm*OhyA) and photoactivated fatty acid decarboxylase from *Chlorella variabilis* NC64 A (*Cv*FAP) into OMVs and transformed oleic acid via a multi-step pathway (Figure 9). While rates were faster for whole cell biocatalysis over OMV biocatalysis (5–6×), when taking into account cell size vs. OMV size, volumetric productivity was >100× higher for OMVs, indicating higher concentrations of enzymes in OMVs over non-hypervesiculating *E. coli* cells. This is the first study on engineering OMVs for biotransformation of unsaturated fatty acids and has opened a new dimension in the field of biotechnology and biomanufacturing.

## 5. Future Directions

Given that directed encapsulation of recombinant enzymes and biomolecules within membrane vesicles such as MVs and OMVs is still in its infancy, there remain multiple challenges, and therefore opportunities, to explore. Most engineered systems rely on the model organism *E. coli*; expanding to other organisms could take advantage of their inherent benefits, but this requires the genetic tools for engineering these organisms. To date, researchers have developed methods to anchor recombinant peptides and proteins such as enzymes both within MV/OMVs and on the exterior surface [17,18,68]. These can include, as referenced above, fusion to ClyA, Hbp, OmpA, INP, and Lpp’, with various scaffolding and tethering methods [17,19,40,41,42,43,44,45,46,47,48,49,50]. However, the efficiency of these methods can vary, and developing design rules for this process could be very beneficial for the field [44]. Additionally, most of these systems have been demonstrated at the lab scale. Developing methods to scale up production of catalytic MVs and OMVs could facilitate their transition to industrial practice.

Despite these challenges, these methods may provide an ideal alternative to whole-cell biocatalysts and some of the inherent issues associated with those systems. Export or “off-loading” of recombinant enzymes in vesicles could circumvent problems of toxicity due to accumulation of recombinant proteins or the catalysts and formation of products that could impact cellular viability. These biological nanoreactors have been shown to be functional in several chemical reactions including biomass degradation [19] and as potential tools for bioremediations directly from bacterial cultures as shown by Alves et al. [17]. These OMVs can be concentrated in a reaction vessel to increase efficiency of product generation or stored for use at a later date or for transport to be used at a location different from that of production. This might be particularly important for the transport of these bioremediation agents to extreme hot or cold environments where recombinant enzymes or whole cell-based catalysts may not work. The advantage of accessibility to substrates would be maintained for surface-anchored enzymes, and the possibility also exists for producing OMVs with target enzymes anchored at their internal surface or contained in their lumen. In this case, use of permeabilization agents to increase substrate access may be less detrimental to OMVs than it would be to whole cells. Overall, use of MVs in biocatalysis has been shown to be a promising avenue for biosynthesis, biosensing, and bioimaging.

## Figures and Tables

**Table 1:** MVs/OMVs naturally involved in biocatalysis.

Organism	Substrate ^1^	Source	Reference
*Fibrobacter succinogenes*	Hemicellulose and pectin	Cow microbiota	[22]
*Clostridium thermocellum*	Crystalline cellulose	Soil	[23]
*Pseudomonas putida*	Lignin	Soil	[24]
*Pseudomonas capeferrum*	Polyurethane compounds	Plastic dump site	[25]
*Bacteroides* sp.	Polysaccharides and proteins	Human microbiota	[26,27]
*Alteromonas macleodii*	κ-carrageenan and red seaweed biomass	Marine	[28]
*Trichoderma reesei*	Cellulose	Fungus/rainforest	[29,30]

^1^ Assumed role for nature is the breakdown of substrate for use as a carbon source. Potential applications for human use would be as breakdown of substrate for use as industrial feedstocks, with the exception of breakdown of polyurethane compounds which could also be used for bioremediation.

**Table 2:** Engineered OMVs in biocatalysis.

Enzyme	OMV Anchor	Application	References
Green fluorescent protein	ClyA	Bioimaging	[40,41]
β-lactamase	ClyA, pectate lyase B signal sequence	Bioremediation	[40,41,45]
Organophosphate hydrolase	ClyA, Ice nucleation protein (INP)	Bioremediation	[40,41,46]
Cellulosome	INP	Biomanufacturing	[19]
Phosphotriesterase	SpyCatcher/SpyTag-OmpA, Lpp’-linker	Bioremediation	[17,44]
DFPase	Lpp’-linker	Bioremediation	[47]
Nanoluciferase	SlyB	Biosensing and Bioimaging	[48,49]
Fatty acid double-bond hydratase and fatty acid decarboxylase	pectate lyase B signal sequence	Biomanufacturing	[50]

## Data Availability

Data sharing not applicable. No new data were created or analyzed in this study. Data sharing is not applicable to this article.
